# Intraarterial Therapies for the Management of Hepatocellular Carcinoma

**DOI:** 10.3390/cancers14143351

**Published:** 2022-07-10

**Authors:** Tushar Garg, Apurva Shrigiriwar, Peiman Habibollahi, Mircea Cristescu, Robert P. Liddell, Julius Chapiro, Peter Inglis, Juan C. Camacho, Nariman Nezami

**Affiliations:** 1 Division of Vascular and Interventional Radiology, Russell H Morgan Department of Radiology and Radiological Science, The Johns Hopkins University School of Medicine, Baltimore, MD 21287, USA; tgarg3@jhmi.edu (T.G.); rliddel1@jhmi.edu (R.P.L.); 2 Division of Gastroenterology and Hepatology, The Johns Hopkins University School of Medicine, Baltimore, MD 21287, USA; ashrigi1@jhmi.edu; 3 Department of Interventional Radiology, University of Texas MD Anderson Cancer Center, Houston, TX 77030, USA; phabibollahi@mdanderson.org; 4 Vascular and Interventional Radiology Division, Department of Radiology, Medical College of Wisconsin, Milwaukee, WI 53226, USA; mcristes@gmail.com; 5 Section of Vascular and Interventional Radiology, Department of Radiology and Biomedical Imaging, Yale University School of Medicine, New Haven, CT 06510, USA; julius.chapiro@yale.edu; 6 Division of Vascular and Interventional Radiology, Department of Diagnostic Radiology and Nuclear Medicine, University of Maryland School of Medicine, Baltimore, MD 21201, USA; pinglis@northwell.edu; 7 Department of Clinical Sciences, College of Medicine, Florida State University, Tallahassee, FL 32306, USA; juan.camacho@radpartners.org; 8 Vascular and Interventional Radiology, Radiology Associates of Florida, Sarasota, FL 34239, USA; 9 Experimental Therapeutics Program, University of Maryland Marlene and Stewart Greenebaum Comprehensive Cancer Center, Baltimore, MD 21201, USA

**Keywords:** hepatocellular carcinoma, transarterial chemoembolization, hepatic artery infusion, selective internal radioembolization therapy, bland embolization, drug-eluting beads–transarterial chemoembolization

## Abstract

**Simple Summary:**

Hepatocellular carcinoma is the most common liver cancer, leading to approximately 700,000 deaths worldwide and 30,000 deaths in the United States every year. Transarterial therapies play a crucial role in the management of these patients, with significant development in techniques over the last couple of decades. The aim of this review is to discuss the different types of transarterial therapies with regards to the pre-procedure, procedural, and post-procedural patient management, along with giving a review of evidence from the literature.

**Abstract:**

Image-guided locoregional therapies play a crucial role in the management of patients with hepatocellular carcinoma (HCC). Transarterial therapies consist of a group of catheter-based treatments where embolic agents are delivered directly into the tumor via their supplying arteries. Some of the transarterial therapies available include bland embolization (TAE), transarterial chemoembolization (TACE), drug-eluting beads–transarterial chemoembolization (DEB–TACE), selective internal radioembolization therapy (SIRT), and hepatic artery infusion (HAI). This article provides a review of pre-procedural, intra-procedural, and post-procedural aspects of each therapy, along with a review of the literature. Newer embolotherapy options and future directions are also briefly discussed.

## 1. Introduction

Image-guided locoregional therapies play a crucial role in the management of patients with hepatic tumors. Transarterial therapies consist of a group of catheter-based treatments where embolic agents or chemotherapeutic agents are directed into the target tumor via their supplying arteries [1,2]. In the current treatment algorithm of HCC, there is a role supporting the use of bland embolization (TAE), transarterial chemoembolization (TACE), drug-eluting beads–transarterial chemoembolization (DEB–TACE), selective internal radioembolization therapy (SIRT), and hepatic artery infusion (HAI) [3]. The physiological principle behind image-guided intraarterial therapies is based on the vascular supply to the tumors. Although the majority of hepatic blood supply is provided by the portal venous system, the hepatic arteries are the predominant blood supply to HCCs. Therefore, selective and local delivery of tumorocidal agents directly into the tumor spares the adjacent hepatic parenchyma and minimizes systemic complications and toxicities [1,2]. Extensive high-quality evidence is available to support the use of image-guided locoregional therapies for the management of hepatic tumors, and this has led to the addition of locoregional procedures to National Comprehensive Cancer Network (NCCN) and Barcelona Clinic Liver Cancer (BCLC) treatment guidelines [4,5]. This article provides a review of pre-procedural, intra-procedural, and post-procedural aspects of each therapy, along with a review of the literature.

## 2. Methodology for Review

We searched the MEDLINE/PubMed database, using the following terms to identify all original research articles published related to TAE, TACE, DEB–TACE, SIRT, and HAI:

((Therapeutic Chemoembolization OR Chemoembolizations, Therapeutic OR Therapeutic Chemoembolizations) OR (radioembolization OR selective internal radiation therapy OR radiation segmentectomy) OR (hepatic arter * infusion OR chemoinfusion) OR (Embolotherapy OR Embolotherapies OR Therapeutic Embolization OR Embolizations, Therapeutic OR Therapeutic Embolizations)) AND (Carcinomas, Hepatocellular OR Hepatocellular Carcinomas OR Liver Cell Carcinoma, Adult OR Liver Cancer, Adult OR Adult Liver Cancer OR Adult Liver Cancers OR Cancer, Adult Liver OR Cancers, Adult Liver OR Liver Cancers, Adult OR Liver Cell Carcinoma OR Carcinoma, Liver Cell OR Carcinomas, Liver Cell OR Cell Carcinoma, Liver OR Cell Carcinomas, Liver OR Liver Cell Carcinomas OR Hepatocellular Carcinoma OR Hepatoma OR Hepatomas) Filters: Clinical Study, Clinical Trial, Clinical Trial Protocol, Clinical Trial, Phase I, Clinical Trial, Phase II, Clinical Trial, Phase III, Clinical Trial, Phase IV, Comparative Study, Controlled Clinical Trial, Meta-Analysis, Multicenter Study, Observational Study, Randomized Controlled Trial, Systematic Review, Technical Report.

The results from this search were reviewed by two authors (T.G. and A.S.) to select the key references to write the review article.

## 3. Treatment Options for Hepatocellular Carcinoma

Given the numerous surgical, medical, and minimally invasive options available for the treatment of HCC, many patients with hepatocellular carcinoma (HCC) benefit when a multidisciplinary approach to treatment is undertaken. This ensures a standardized workup and access to the most up-to-date treatment options based on individual patient parameters, including social factors; underlying liver function; tumoral factors, such as tumor size; invasion of blood vessels and extrahepatic structures; transplant eligibility; institutional experience; and access to various therapies.

### 3.1. Surgical and Systemic Therapies

Surgical resection and liver transplantation are classically considered to be the only potentially curative treatment options for HCC. Partial hepatectomy is only offered to patients with solitary HCC without any evidence of macrovascular invasion or cirrhosis, and without significant stigmata of portal hypertension, while transplantation is reserved for patients with early HCC within the United Network for Organ Sharing (UNOS) criteria, an adapted version of the Milan criteria [6]. In patients who meet the stringent criteria for future transplantation, “bridging” therapy can prevent disease progression and reduce the dropout rate from the transplant waiting list [7]. Locoregional therapies such as radiofrequency ablation, microwave ablation, TAE, TACE, DEB–TACE, and SIRT have all been shown to be useful as bridge therapies [8,9,10,11,12,13,14,15,16,17,18,19]. “Downstaging” therapies are used in cases of advanced HCC in patients without distant metastasis to reduce the intrahepatic tumor burden [7,20,21]. Locoregional therapies are commonly used for this purpose, allowing many previously ineligible patients to become eligible for liver transplantation under the Milan or UNOS criteria [22]. These therapies have been shown to improve the 1- and 5-year survival after transplant compared to liver transplantation alone [23].

For patients with advanced HCC, systemic therapy can be considered as the first-line treatment [24,25]. Currently, the United States Food and Drug Administration (FDA) has approved six agents for use as systemic therapies. These include atezolizumab + bevacizumab, sorafenib, lenvatinib, regorafenib, cabozantinib, ramucirumab, and nivolumab [26,27,28,29,30].

### 3.2. Locoregional Therapies

According to the BCLC algorithm, the choice of locoregional therapy in patients with early stage HCC is based on the size of the tumor, underlying liver disease, and liver function. Locoregional therapies can generally be subdivided into (1) percutaneous and (2) intraarterial modalities. Generally, ablative techniques are used in tumors less than ≤3 cm located remotely from the hilar bile ducts [31,32,33,34,35,36]. Ablation involves the direct application of thermal or nonthermal/chemical energy into tissues in order to induce cellular injury and apoptosis. The most commonly utilized ablation technologies within the liver include radiofrequency ablation, microwave ablation, cryoablation, and percutaneous ethanol injection.

**Figure 1:**
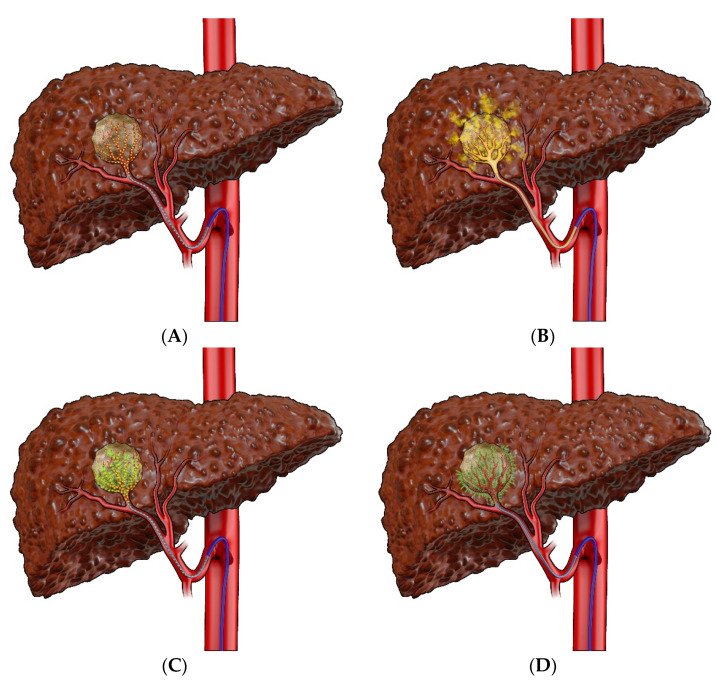
Transarterial therapies. (**A**) Bland embolization (BE). (**B**) Conventional transarterial chemoembolization (cTACE). (**C**) Drug-eluting beads–transarterial chemoembolization (DEB–TACE). (**D**) Radioembolization.

Transarterial therapies rely on the arterial blood supply of targeted tumors and include TAE, TACE, DEB–TACE, SIRT, and HAI (Figure 1). These techniques are generally considered when treating larger tumors, multifocal disease, or for tumors centrally located or adjacent to major vasculature/bile ducts. The efficacy of TAE stems from a reduction of blood flow to the supplying artery, thus leading to ischemia in the region of the tumor that can then lead to tissue necrosis. With TACE and DEB–TACE, a high concentration of chemotherapy is additionally delivered to tumor cells [37], whereas the mechanism of action for SIRT is the emission of high-dose beta-radiations into the capillary bed of the tumor [3,38]. In HAI, chemotherapy is administered non-selectively to the liver and embedded tumors via the proper hepatic artery.

Radiation therapies are also considered locoregional therapies in patients with inoperable or unresectable HCC. The two commonly used types of radiation therapy are external beam radiation therapy (EBRT) and stereotactic body radiation therapy (SBRT). In EBRT, a high dose of radiation is delivered to the target tissue (liver tumor), while a lower dose is delivered to the normal liver parenchyma surrounding the tumor. SBRT is a more advanced version of EBRT which helps to deliver a larger amount of ablative dose to the target tissue (liver tumor) without further increasing the dose delivered to the normal liver parenchyma [39,40]. Proton beam therapy (PBT) is another radiation therapy option in which a finite range of a radiation dose is delivered to the target tissue (liver tumor); however, it causes lower delivery of radiation to the normal liver tissue at the margins, providing a dosimetric advantage compared to SBRT [41]. Evidence from multiple studies in the literature has shown that SBRT is useful in the treatment of patients with unresectable, locally advanced, or recurrent HCC [42,43,44,45]. EBRT and SBRT are currently not a part of BCLC algorithm for treatment of the HCC. An in-depth discussion of radiation therapies is beyond the focus of this review article.

## 4. Indications and Patient Selection for Treatment with Transarterial Therapies

**Figure 2:**
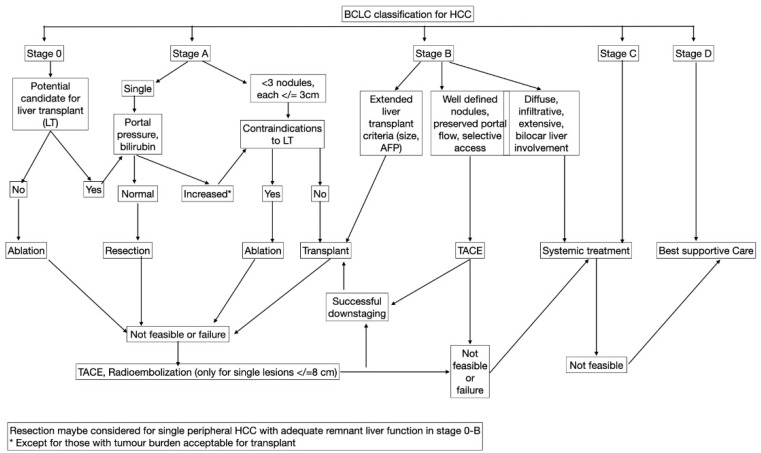
Indications for the use of transarterial therapies in patients with hepatocellular carcinoma (HCC).

Transarterial therapies can be used to reduce the burden of tumor within the transplant criteria (downstaging), to control the growth of tumor in patients who are currently on the transplant waiting list (bridging), and to increase the survival of patients who are not eligible to undergo a transplant (palliative). A multidisciplinary tumor board consisting of oncologists, surgeons, diagnostic and interventional radiologists, and hepatologists optimally can decide in aggregate if transarterial therapy is indicated for each individual patient [46]. Figure 2 shows the BCLC-approach indications for transarterial therapies in HCC [5].

## 5. BCLC Staging and Predictors of Response

The BCLC scoring system classifies patients into five stages (BCLC 0, BCLC A, BCLC B, BCLC C, and BCLC D; Table 1) on the basis of size and extent of the primary HCC lesion, performance status of the patient, and presence of vascular invasion and extrahepatic disease [5]. In some studies, the BCLC scoring system has been shown to outperform other prognostic scoring systems in patients undergoing surgical treatment [47,48]. Patients with very early and early BCLC grades have a low tumor burden (≤3 tumors, <3 cm), preserved liver function (Child–Pugh A), and are fully active (ECOG 0). These patients have been shown to have a life expectancy of more than 5 years. Patients are classified into intermediate stage (BCLC B) if they have a multinodular tumor with preserved liver function (Child–Pugh A to B7) and normal functional status (ECOG 0). Advanced stage (BCLC C) includes patients with macrovascular invasion (i.e., tumor thrombus) or extrahepatic disease, along with preserved liver function and worsening performance status (ECOG 1 and 2). The life expectancy of patients in this group has been shown to be approximately 10 months. Terminal-stage disease (BCLC D) includes patients with any tumor burden but with the presence of poor liver function (Child–Pugh C) or a poor performance status (ECOG >2). BCLC-D patients have a life expectancy of only 3 months [5]. The presence of liver decompensation (ascites, jaundice, and encephalopathy) is considered as non-preserved liver function regardless of the Child–Pugh and model for end-stage liver disease (MELD) score. The BCLC staging system has been adopted widely due to its simplicity and prognostic reproducibility. However, the BCLC system has been shown to be conservative in terms of intermediate- and advanced-stage management.

### 5.1. Eastern Cooperative Oncology Group (ECOG)

The ECOG Performance Status Scale is used to assess a patient’s level of function by looking at his/her ability to perform physical activities, complete daily activities, and take care of him/herself. The ECOG Performance Status Scale is an essential component of BCLC classification. Table 2 shows the ECOG Performance Status Scale [49].

### 5.2. Child–Pugh Classification

Based on clinical and laboratory information, Child–Pugh scoring predicts the prognosis of patients with cirrhosis by estimating their liver function. Table 3 demonstrates the Child–Pugh classification of severity of cirrhosis. Patients with a score of 5 to 6 are classified in class A and are said to have well-compensated cirrhosis, those with a score of 7–9 are classified in class B and are said to have significant functional compromise of the liver, and those with a score of > 9 are classified in class C and are said to have decompensated cirrhosis [50]. According to several studies, TACE is recommended for Child–Pugh class A and highly selective class B cirrhosis [51].

### 5.3. Albumin–Bilirubin (ALBI) Score

A significant limitation of the Child–Pugh classification is its subjective assessment due to the inclusion of ascites and encephalopathy in the score that can easily be modified by medication(s). Therefore, a new score called ALBI was developed that includes only objective parameters, serum albumin and bilirubin, to assess the liver function. The formula for calculating the ALBI score is as follows:

ALBI score = (log_10_ bilirubin 0.66) (albumin 0.085)

where bilirubin is in mmol L^−1^, and albumin is in g L^−1^.

Table 4 shows ALBI grading according to the ALBI score. Child–Pugh class A can be subdivided into ALBI Grades 1 and 2 [52]. According to several retrospective studies, ALBI is superior to the Child–Pugh classification in regard to prognosis identification, accuracy, and stratification [53]. The ALBI score has shown an excellent prognostic and predictive ability for patients undergoing palliative transarterial therapies [54]. It has also been used to categorize patients belonging to BCLC-B class undergoing TACE [55].

### 5.4. Platelet–ALBI (pALBI) Score

In order to include the presence of paraneoplastic syndrome in patients with HCC, the pALBI score was developed. The pALBI score includes platelet counts as an indicator of a paraneoplastic syndrome of HCC. The formula for the pALBI score is given below:

PALBI score = 2.02 × log_10_ bilirubin − 0.37 × (log_10_ bilirubin)^2^ − 0.04 × albumin − 3.48 × log_10_ platelets + 1.01 × (log_10_ platelets)^2^

Table 5 shows the pALBI grading according to the pALBI score [56]. According to a study by Liu et al., a higher C-index was seen for the PALBI grade than the ALBI grade, thus indicating its usefulness as a robust statistical model that provides improved discriminatory power [57].

### 5.5. The Neutrophil-to-Lymphocyte Ratio (NLR)

In patients with inflammation, neutrophils have been shown to reduce the activity of different types of lymphocytes. The ratio of neutrophils to lymphocytes can be used as a marker of inflammation and immune response [58]. A couple of studies have highlighted that a higher lymphocyte count correlates with treatment response and prognosis [59,60]. As per the study conducted by Wang et al., NLR values increased three days post-TACE and then fell back to baseline after one month; a higher NLR (>2.4) was associated with overall survival of 15.6 months vs. a lower NLR (≤2.4) having an overall survival of 27.1 months [61].

### 5.6. MELD Score

The MELD score was first developed to predict the survival of patients with liver cirrhosis who underwent placement of a transjugular intrahepatic portosystemic shunt for their disease management. Currently, the MELD system is used to rank liver-transplantation candidates’ priority. The MELD score is calculated by measuring three objective variables: total bilirubin, creatinine, and INR [62]. In a study by Sawhney et al., a MELD score of higher than ten is associated with higher mortality after TACE (Hazard ratio: 4.9, *p* = 0.001) [63].

### 5.7. Assessment for Retreatment with Transarterial Chemoembolization (ART) Score

The ART score is used to predict the survival of patients with HCC after their first TACE treatment by looking at the increase in Child–Pugh score (1.5 point for 1 point increase, 3 points for ≥2 points increase, and 0 points for no increase), presence or absence of radiological response (1 point for no response, and 0 points for presence of response), and elevation of aspartate transaminase (4 points for >25% increase, and 0 points for ≤25% increase) [64]. A high ART score after the first TACE was found to be associated with the risk of major adverse events upon the second TACE (*p* = 0.011) [64]. When the patients where stratified on the basis of their ART score in the initial validation study, patients with a score of 0–1.5 points were found to have a median OS of 23.7 months, and those with ≥2.5 points were found to have a median OS of 6.6 months [64]. These findings were confirmed in an unrelated study conducted by Abbasi et al. [65].

### 5.8. ASARA Scoring System

More recently, the ASARA scoring system, a predictive model for stratifying candidates based on liver function for suitability for initial/repeated TACE procedure, has come into the picture. AFP ≥ 400 ng mL^−1^, tumor size (maximum diameter >7 cm), increase in ALBI score, no tumor response, and increase in AST ≥25% are each assigned a score of 1 (total score range of 0–5) [66]. The median OS for patients with a score of ≤2 was significantly higher than those with a score of >2 (*p* = 0.006). This model suggests that a score of >2 predicts TACE failure; thus, switching to systemic therapy should be considered in such scenarios [66]. For patients with Child–Pugh class B and impaired hepatic function, the modified AS (ARA) model could be applied to screen individuals for initial TACE treatment (cutoff value >1 predicts worse prognosis, *p* = 0.004) [66].

### 5.9. Hepatoma Arterial-Embolization Prognostic (HAP) Score

The HAP scoring system stratifies patients into low (HAP class A), intermediate (HAP class B), high (HAP class C), and very high risk (HAP class D) groups for predicting survival after TACE [67]. An albumin level of <36 g dL^−1^, AFP levels of >400 ng mL^−1^, bilirubin levels of >17 μmol L^−1^, and a maximum tumor diameter of >7 cm are each given 1 point in this scoring system. The OS for class A (score 0), class B (score 1), class C (score 2), and class D (≥2) were 25.5, 18.1, 8.9, and 5.9 months, respectively, in the validation model of this scoring system [67]. According to a few studies, HAP classification might be a better predictor of survival than Child–Pugh, CLIP, BCLC, and MELD scoring systems [67,68].

### 5.10. Selection for Transarterial Chemoembolization Treatment (STATE) Score

The STATE score has been developed to identify unbefitting patients for the initial TACE session. The STATE score = serum albumin (9g L^−1^) − 12 (if up-to-7-out) − 12 (if CRP >1 m dL^−1^). Patients with a total score of <18 points had a poorer prognosis as opposed to patients with a score of ≥18 (median OS: 5.3 months vs. 19.5 months, *p* < 0.001) [69]. Additionally, patients with a score of <18 points had a significantly greater rate of serious adverse events following their initial TACE session and higher short-term mortality (number needed to harm = 4) [69]. These findings have been confirmed in a few other studies [68,70].

### 5.11. Aspartate-Aminotransferase-to-Platelet Index (APRI) Score

The APRI scoring system was developed by Wai et al. for predicting significant fibrosis and cirrhosis in patients with chronic hepatitis C [71]. Lately, the APRI score has been used to predict survival in patients with HCC. A meta-analysis suggested a significant association between high APRI levels and poor prognosis in patients with HCC (HR 1.59, *p* < 0.001) [72]. Furthermore, another study indicated that the APRI score has indistinguishable accuracy but higher negative predictive value and sensitivity for predicting post-TACE acute liver function deterioration when compared to Child–Pugh scoring [73].

### 5.12. Tumor Size, Tumor Number, Baseline AFP, Child–Pugh Class, and Objective Radiological Response (SNACOR) Score

The SNACOR classification is another predictive model for the prognostication of patients with HCC who underwent TACE. Tumor size (<5 cm = 1 point, ≥5 cm = 2 points), tumor number (<4 = 1 point, ≥4 = 2 points), baseline AFP levels (<400 ng mL^−1^ = 1 point, ≥400 ng mL^−1^ = 2 points), Child–Pugh class (A = 1 point, B = 2 points), and response evaluation (complete response + partial response = 1 point, stable disease + progressive disease = 2 points) are the components of this scoring system. The patients are classified into three groups based on their total score: low-risk (0–2), intermediate (3–6), and high-risk (7–10) groups [74]. This model suggested that the patients in the intermediate (HR 2.13) or high-risk (HR 6.17) groups had a significant risk of death compared to the low-risk group (*p* < 0.001) and might not be candidates for retreatment with TACE [74]. A validation study described this model as adequate to distinguish favorable vs. impaired prognosis groups with moderate performance [75].

### 5.13. Okuda Score

The Okuda prediction model, one of the oldest staging systems, is a scoring scheme based on tumor size, ascites, serum bilirubin, and serum albumin levels; each scored 0/1. Based on the total score, patients are classified into three stages: Stage I (0 points), Stage II (1–2 points), and Stage III (3–4 points) [76]. In the original cohort of 850 patients diagnosed with HCC in 1975–1983, the median OS between the three stages was 11.5 months (Stage I) vs. 3 months (Stage II) vs. 0.9 months (Stage III) [76]. However, Levy et al. found no significant difference in the median OS between Stage II and Stage III patients (3.2 months vs. 3.1 months, *p* = −0.43) in their cohort of patients [77].

### 5.14. Cancer of the Liver Italian Program (CLIP) Score

The CLIP scoring system considers the residual liver function and tumor characteristics for the prognostic assessment of HCC patients [78]. In a study by Li et al., patients with a CLIP score of 0–2 were found to have a longer OS than those with CLIP scores of 3–5 (13 months vs. 4 months, *p* = 0.001), making them better candidates for TACE [79]. A recent study suggests that the CLIP system is superior for predicting survival in patients with unresectable HCC treated with TACE compared to the Okuda classification system and is easier to implement [68].

## 6. Patient Preparation

### 6.1. Pretreatment Imaging

A contrast enhanced multiphasic MDCT or MRI can be used for the diagnosis of HCC in lesions >1 cm and should be performed preferably within three months of the procedure [80]. Imaging should be obtained to assess factors such as vascular anatomy, lesion size, number of lesions, macrovascular invasion, and extrahepatic spread, which help determine eligibility for locoregional and/or systemic therapies [81]. MDCT is considered superior for delineating the vascular anatomy of the hepatic vasculature, as well as identifying parasitic tumor feeders; however, MRI demonstrates superior evaluation of lesion enhancement, morphology, and classification [82].

### 6.2. Pre-Procedural Labs and Tumor Markers

The following tests are recommended for diagnosing the extent of liver function and obtaining baseline liver function for potential postprocedural hepatotoxicity [46]:

- Aminotransferase,
- Cholinesterase,
- Alkaline phosphatase,
- Gamma-glutamyl transferase,
- Bilirubin,
- Albumin,
- Prothrombin time,
- Creatinine,
- Electrolytes.

In addition to the above tests, testing for tumor markers is also essential. Alpha-fetoprotein is the most commonly used tumor marker for HCC, as it is able to predict disease prognosis and aids in monitoring tumor recurrence [83,84]. More recently, vitamin K absence, antagonist-II (PIVKA-II), has shown to increase HCC detection rates and can be obtained in adjunct to AFP and imaging [85].

### 6.3. Pre-Procedural Preparation

Fasting for six to eight hours is required for preparation for conscious sedation or general anesthesia.

Proton pump inhibitors may be given to reduce the chances of post-procedure gastritis/duodenitis and mitigate the significance of non-target embolization. Dexamethasone (20 mg intravenously) may also be given to reduce the risk of post-embolization syndrome. Intravenous fluids should be considered in those without cardiac contraindications. Anxiolytics have also been found to be beneficial pre-procedure [46]. If predisposing factors for liver abscess listed below are found, then an antimicrobial regimen such as 400 mg of moxifloxacin by mouth daily, beginning three days pre-procedure to seventeen days post-embolization, should be prescribed; however, longer courses are also utilized [86].

- Biliary tree canulation/dilation/sphincterotomy;
- Presence of biliary prosthesis (e.g., plastic and metallic stents);
- Presence of a bilioenteric anastomosis;
- Presence of TIPS [87];
- Biliary or gallbladder stone.

Patients without the above risk factors are typically given a single dose of cefazolin immediately prior to the procedure. The pre-procedural target international normalized ratio (INR) and platelet values are desired to be INR ≤2–3 and a platelet count of >20,000 μL^−1^, as, according to the Society of Interventional Radiology Consensus Guidelines, these procedures are classified to be low risk for bleeding [88].

## 7. Techniques: Angiography and Embolization Technique

### 7.1. Initial Angiography

Either the common femoral artery (CFA) or radial artery (RA) access can be used for performing transarterial embolization [89]. A 4–6 Fr vascular sheath is used with either approach. If the RA access is selected, a cocktail of heparin and one or more vasodilators (nitroglycerin, verapamil, or nicardipine) is generally administered intraarterially via the sheath once access has been achieved [90].

Mesenteric angiography is performed to visualize and define the relevant vascular anatomy and identify tumoral arterial supply, as well as collaterals and arterial supply at risk of non-target embolization [91]. A superior mesenteric angiogram is obtained first to exclude aberrant vascular anatomy and confirm portal vein patency on delayed imaging. Then a celiac arteriogram is performed to identify target (hepatic artery branches) and non-target arteries and hypervascular tumors [92]. In situations where, extrahepatic arterial recruitment is suspected, extrahepatic vessels (phrenic, gastric, and internal mammary) are also evaluated [93]. Frequently, intraprocedural cone-beam CT technology is utilized to identify and/or confirm arterial supply of targeted tumors [94].

Once the procedure is completed, all the catheters and sheaths are removed, and hemostasis is achieved by either manual compression or with the help of a closure device [95].

### 7.2. Embolization Techniques

#### 7.2.1. Bland Embolization (TAE)

In TAE, after following the steps described in the previous section, lobar embolization or subselective embolization can be performed depending on the arterial distribution, location of the tumor, and tumor burden. TAE is usually performed by using embolic agents ranging from 40 to 120 µm in size that may be spherical or non-spherical, resorbable or non-resorbable, calibrated or non-calibrated [96,97]. Particles are mixed with iodinated contrast, normal saline solution, and an antibiotic in some cases. Embolization is performed to the endpoint of stasis of flow in the target vessel(s).

#### 7.2.2. Transarterial Chemoembolization (TACE)

**Figure 3:**
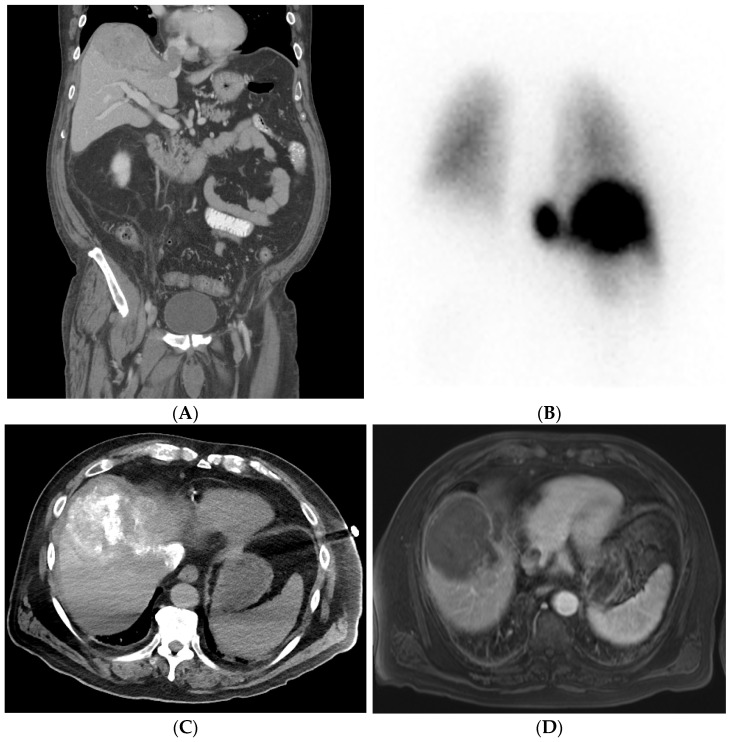
Patient with right lobe HCC with extension into the hepatic vein causing thrombosis. Pre-procedural investigation showed 30% shunt fraction, and therefore the patient was treated with cTACE. (**A**) Pre-procedural coronal CT shows right lobe HCC lesion with hepatic vein thrombosis. (**B**) A 99mTc-MAA SPECT/CT shows a lung shunt with a lung shunt fraction of 30%. (**C**) Intraoperative CT showing good lipiodol uptake in the treated lesion areas. (**D**) Follow-up MRI showing reduced non-enhancing HCC and hepatic vein thrombus due to good response after treatment.

**Figure 4:**
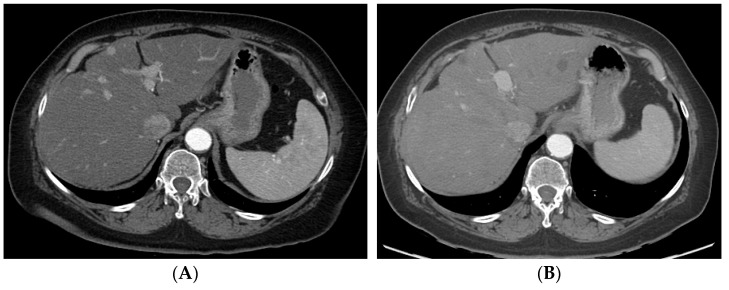

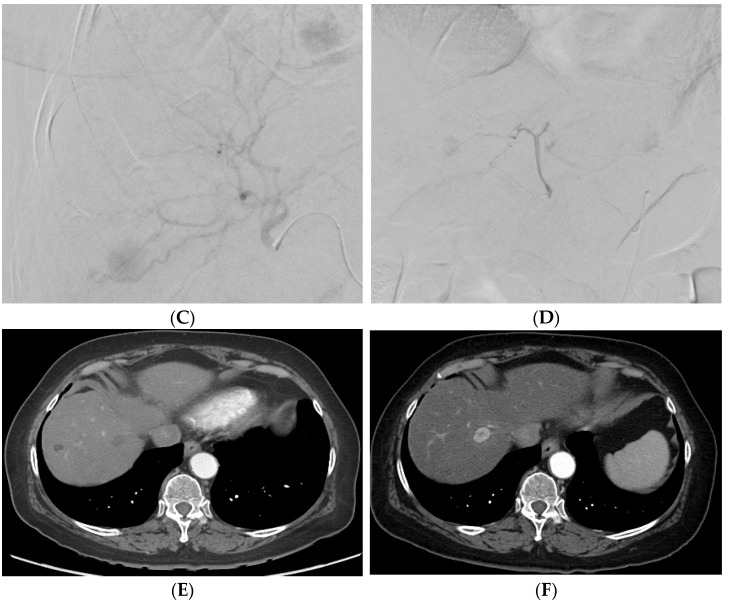
A patient with multifocal HCC who underwent DEB–TACE for its management. CT showing multifocal HCC involving the right (**A**) and left lobe (**B**). Common hepatic artery angiogram showing tumor blush (**C**), which completely disappeared after DEB–TACE (**D**). Follow-up CT showing good tumor response in the right (**E**) and left (**F**) lobe of the liver.

In TACE, following the above steps, a microcatheter is used to subselectively catheterize target branches. The goal of subselective catheterization is to maximize the delivery of chemoembolic material to tumor, while minimizing, as much as possible, delivery to the normal liver. Once the catheter position is optimized, conventional or DEB–TACE can be performed under real-time fluoroscopic observation. For conventional TACE, the prescribed chemotherapy is combined with ethiodized oil and contrast and then continuously administered until the peritumoral venous plexus is visualized. However, this approach might be difficult to achieve in big tumors. For DEB–TACE, the endpoint of administration is sub-stasis of flow within the supplying vessel. Caution must be used to prevent over-embolization, resulting in reflux into non-target liver and non-target organs (i.e., bowel, pancreas, and spleen). In cTACE, particles or gelfoam are often delivered after chemoembolic material to prevent “washout” of chemotherapy. If multiple tumors are present, the best approach is to target each individual tumor as selectively as possible. Classically, only one lobe (right or left) is treated in a single setting; however, this approach is variable with the ability to perform subselective targeted delivery of chemoembolic material. If lipiodol or radiodense beads are utilized, non-contrast cone-beam CT or non-contrast CT can be obtained post-delivery to visualize the extent of lipiodol deposition. Figure 3A–D shows CT and MRI of a patient who underwent cTACE. Figure 4A–F shows the CT and angiograms of a patient who underwent DEB–TACE.

#### 7.2.3. Selective Internal Radiation Therapy (SIRT)

**Figure 5:**
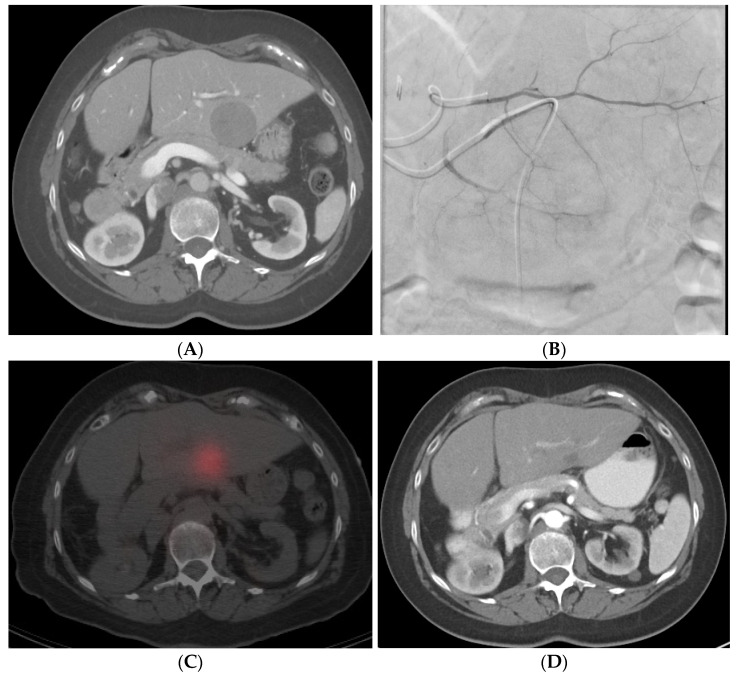
Radiation segmentectomy in a patient with HCC with a past surgical history of right hepatectomy. (**A**) CT showing a large lesion in the left lobe of the liver. (**B**) Left hepatic artery angiogram showing vessels supplying the tumor. (**C**) SPECT-CT imaging after left segment-2 sub-segmentectomy, showing good dose delivery. (**D**) Follow-up CT showing good response in the treated area.

With SIRT/Y-90-radioembolization, the added step of technetium-99 m macroaggregated albumin (Tc-99 m MAA) shunt study, or “mapping”, prior to the SIRT is necessary. Following diagnostic angiography and subselective catheterization of supplying tumoral feeders, approximately 4 mCi of Tc-99 m MAA is delivered into the lobe or segment containing the target tumor. The target artery may be identified with the aid of cone-beam CT. After the injection of Tc-99 m MAA, the patient is taken for planar nuclear imaging or SPECT/CT in order to assess for any shunting (specifically to the lung) and assess technical success by visualizing Tc-99 m MAA deposition within targeted tumors. The patient then returns for Y-90 delivery 1–4 weeks following the shunt study (although “same-day” mapping and delivery are also performed at select centers). The prescribed dose of Y-90 depends on multiple factors such as tumor size and number, liver function, and the microparticle of choice. Y-90-radioembolization is performed from the same catheter position utilized for the Tc-99m MAA shunt study. SPECT/CT is then again sometimes obtained post-procedure to assess Y-90 distribution and dose delivery to the targeted tumor. Figure 5A–D shows the CT, SPECT, and angiogram of a patient who underwent segment-two segmentectomy.

#### 7.2.4. Hepatic Artery Infusion (HAI)

In hepatic artery infusion (HAI), chemotherapy is delivered to the tumor by infusing it into the hepatic artery by placing either a surgically implanted pump/port or by placing a percutaneous catheter, which can be connected to an external pump [98,99,100]. For percutaneously placed catheters, the Seldinger technique is used to access the artery and place infusion catheters. Left subclavian and right femoral artery accesses are frequently used. The most commonly used site for access is the common femoral artery, as it is easier to access due to its superficial and less torturous course. In the “fixed-catheter-tip” technique, the distal tip of the catheter is fixed to the gastroduodenal artery, and the chemotherapy is infused to the tumor with the help of a side hole which is positioned into the proper hepatic artery [101]. Another technique which can be used is the “long tapered catheter placement” technique [102]. In this technique, the catheter is positioned distally into the segmental hepatic artery, and the side hole of the catheter is placed at the origin of the proper hepatic artery [103].

## 8. Mechanism of Action of Transarterial Therapies

### 8.1. Bland Embolization (TAE)

While the portal vein and hepatic artery offer duality in terms of blood supply, HCC generally relies on hepatic arteries to meet oxygen requirements. TAE involves delivering embolic particles into the arterial supply of targeted tumors, thereby creating hypoxia and subsequent infarction seen on follow-up contrast enhanced imaging (CT or MRI) [104]. TAE is similar to other catheter-directed embolic interventions, such as DEB–TACE or cTACE, but may be used in patients in whom chemotherapy is contraindicated or if chemotherapeutic agents are unavailable.

### 8.2. Transarterial Chemoembolization (TACE)

Delivering embolic particles within a tumor’s vascular bed results in localized tissue hypoxia; however, the concurrent addition of chemotherapy may have additive antitumor effects [104]. An animal study combining cTACE with radiotherapy has shown promising results; this two-pronged approach could potentially help target cancer cells not reached by Y-90 beads due to arterial inflow limitation [105]. TACE is commonly used in cases of unresectable HCC and is first-line therapy for patients with intermediate-stage HCC [106]. Conventional TACE is referred to as Lipiodol^®^-based TACE. The two-part process begins with transcatheter delivery of chemotherapy, followed by particle embolization. The most common chemotherapeutic agents include cisplatin (100 mg), doxorubicin (50 mg), and mitomycin C (10 mg). These agents are mixed with lipiodol (Ethiodol) in a 1:1 to >5:1 (lipiodol:chemotherapy) volume ratio [107]. A higher mixing ratio (larger volume of iodized oil) ensures the creation of a water-in-oil-type emulsion. A water-in-oil-type emulsion is advantageous in the selective delivery of an anticancer drug to the tumor. Moreover, cTACE may be repeated serially when there is clear evidence of a viable tumor, with some studies recommending a minimum of three sessions before abandoning cTACE in patients with nonresponding intermediate-stage HCC [108].

### 8.3. Drug-Eluting Beads–Transarterial Chemoembolization (DEB–TACE)

With the advent of chemotherapy-loaded embolic spheres, an additional modality of chemotherapy delivery became available. Drug-eluting beads dually embolize selective tumor-feeding vessels and deliver chemotherapy in a concentrated fashion [106]. In this sense, DEB–TACE is different than conventional TACE, as the previously described two-part process (cTACE) is exchanged for a single-step modality (DEB–TACE). In randomized trials comparing cTACE and DEB–TACE, the major difference were less post-procedural pain and shorter hospital admission in DEB–TACE, this finding can be explained by the deeper penetration of chemoembolic material into the liver capsule and peritumoral portal venous system with cTACE [109].

### 8.4. Selective Internal Radiation Therapy (SIRT)

Radiation therapy has been a staple of oncologic therapy, and its usage has typically been reserved for external beam radiation. However, the collateral damage to surrounding parenchyma limits its use in patients with already tenuous liver function. The localized delivery of radiation-emitting microspheres is a means by which to decrease this collateral damage. The most commonly used radioisotopes include yttrium-90 (Y-90) and Holmium-166 (166-HO). SIRT is effective in cases of unresectable tumors or chemotherapy-resistant disease. SIRT, specifically Y-90, has proven to be efficacious in downstaging HCC for purposes of transplant eligibility [110]. Furthermore, 166-HO is a more recently isolated radioisotope and has additional benefits beyond Y-90. Most interesting, perhaps, is that 166-HO microspheres emit high-energy beta particles, in addition to gamma radiation, therefore offering single-photon emission computed tomography compatibility. Moreover, 166-HO microspheres are also paramagnetic and therefore hyper-resonant on MRI (magnetic resonance imaging). SIRT is delivered via two approaches: lobar/sub-lobar and segmentectomy. The lobar/sub-lobar approach is reserved for instances of multifocal disease, whereas a segmentectomy is chosen in cases of solitary tumors measuring 5 cm or less and that are accessible angiographically.

Currently, two manufactured agents are utilized for SIRT: glass beads and resin beads. Glass beads (TheraSphere, Boston Scientific, Marlborough, MA) are 20–30 microns in size and deliver a large activity to the tumor bed (~2500 Bq). However, resin beads (SIR-Spheres, Sirtex, Woburn, MA, USA) are larger in size (20–60 microns) and deliver a lower level of activity to the tumor bed (50 Bq) but produce a greater degree of embolization [111]. The recommended delivery dose for glass beads is a tumoral dose of 400 Gy for successful radiation segmentectomy and 150 Gy for resin bead; however, dosimetry data are continuing to evolve rapidly and are decided based on personalized dosimetry in most cases [112,113].

### 8.5. Hepatic Artery Infusion (HAI)

HAI is a widely employed alternative to systemic chemotherapy, as it directly delivers chemotherapeutic agents to the tumor feeding vessels and reduces the systemic toxic side effects of these drugs through the first-pass effect of the liver [114]. In a recently conducted phase-three study for large unresectable HCC without macrovascular invasion, comparing HAI with FOLFOX (fluorouracil, leucovorin, and oxaliplatin) vs. TACE, HAI with FOLFOX (median overall survival 13.3 months) showed a significantly improved overall survival rate as compared to TACE (median overall survival of 10.8 months). The infusion was performed once every three weeks for up to six courses [100]. A study comparing treatment with sorafenib alone vs. sorafenib combined with FOLFOX HAI (soraHAIC) reported a tolerable safety profile and improved efficacy with soraHAIC (median overall survival 13.37 months) vs. sorafenib alone (median overall survival of 7.13 months) [115]. The SILIUS trial consisting of 206 patients compared sorafenib alone vs. sorafenib plus low-dose cisplatin and FU HAI. The study found no significant survival benefit from combining sorafenib with low-dose cisplatin and FU (median overall survival with sorafenib alone was 11.8 months vs. overall combination survival of 11.5 months) [116]. The use of sorafenib with HAI compared to sorafenib along with HCCs with major portal vein tumor thrombus was recently studied in a randomized controlled trial which showed a better median overall survival, higher objective response rate, and longer median progression-free survival in the sorafenib with HAI group [117]. However, the survival benefit of combining sorafenib with HAI has largely been shown to be variable, thus necessitating more studies.

## 9. Contraindications to Transarterial Embolization

### 9.1. Bland Embolization (TAE) and Transarterial Chemoembolization (TACE)

There are no absolute contraindications to TAE or chemoembolization procedures; however, the presence of various factors can make patient candidacy unfavorable to undergo these procedures. Relative contraindications include contraindications to arteriography; absence of portal venous blood flow; extensive tumor with replacement of both the lobes of liver; decompensated cirrhosis, which is indicated by Child–Pugh class C, or Child–Pugh class B score >8, along with jaundice; clinically overt hepatic encephalopathy, refractory ascites, and/or hepatorenal syndrome; tumor size more than 10 cm; active cardiovascular or lung disease; untreated gastroesophageal varices at the risk of bleeding; bile duct occlusion or incompetent papilla; poor performance status; active systemic infection; contraindications to chemotherapy; or poor tolerance to prior procedures [81,92,118,119].

### 9.2. Selective Internal Radiation Therapy (SIRT)

Contraindications to SIRT include the history of prior liver irradiation, significant liver dysfunction/decompensation, metastasis to organs other than the liver, pregnancy, irregularities in hepatic venous anatomy that preclude radioembolization, capecitabine treatment three months before the procedure, pathological shunt fraction causing a lung dose of ≥30 Gy in a single application or presence of flow to the gastrointestinal tract from the arterial supply of the tumor that cannot be corrected by transcatheter techniques, and abnormal laboratory values. Although not absolute guidelines, a white blood cell count less than 2500 cc^3^, neutrophil count less than 1500 cc^3^, platelet count less than 60,000 cc^3^, alanine transaminase or aspartate transaminase more than five times the normal value, bilirubin more than 2 mg dL^−1^, albumin less than 3 mg dL^−1^, and creatinine more than 2.5 mg dL^−1^ have been proposed as limits to therapy [120].

## 10. Post-Procedural Follow-Up

The post-procedural follow-up after the embolization procedure is similar for many patients regardless of the type of transarterial therapy they are receiving. The follow-up includes a clinic visit to reassess the performance status of the patient and ensure symptomatic management; the laboratory tests include a comprehensive metabolic panel, complete blood count, international normalized ratio, normal tumor markers, and multiphasic contrast enhance MRI or CT of the abdomen with intravenous iodine-based contrast for follow-up. Follow-up after liver-directed therapy is recommended at 1, 3, 6, and 12 months in the first year after the procedure and every 6 months thereafter. Of note, post-treatment imaging of HCC following radioembolization is recommended at 6–8 weeks following delivery, as the localized post-radioembolization parenchymal enhancement imitates the treated disease, making response difficult or impossible to assess before these changes evolve. Additionally, the follow-up schedule should be tailored for each patient according to their treatment goals, transplant status, local imaging availability, etc. [121].

## 11. Complications

### 11.1. Bland Embolization (TAE) and Transarterial Chemoembolization (TACE)

The most common complication associated with embolization is postembolization syndrome, which can be seen in up to 90 percent of patients and can present with fatigue, right upper quadrant pain, low-grade fever, nausea, vomiting, and ileus [122,123,124,125]. Symptoms due to the post-embolization syndrome are generally self-limiting, and recovery is often seen within 7 to 10 days [126]. Other less common but serious complications associated with embolization are most often related to treatment-induced ischemic damage, leading to liver failure [127,128,129,130], hepatic abscess [131], acute cholecystitis, biliary duct injury [130,132], gastroduodenal ulceration [126,130], renal dysfunction [130], pulmonary lipoid embolization [133,134], cerebral lipoid embolization [135], interstitial pneumonia [136], tumor lysis syndrome [130,137], and risk of reactivation of hepatitis B infection [138,139].

### 11.2. Selective Internal Radiation Therapy (SIRT)

Similar to cTACE, post-embolization syndrome is the most common complication associated with SIRT; however, it is less severe in patients undergoing SIRT, and the incidence is lower [140,141,142,143]. Other complications associated with SIRT include hepatic dysfunction, which can manifest as liver failure; radiation-induced liver disease (RILD), which can be seen in 5 to 23 percent of patients [141,144]; hepatic fibrosis [145]; portal hypertension [145,146]; radiation pneumonitis in less than 1 percent of patients [141]; gastric or duodenal injury in less than 5 percent of patients; and lymphopenia [147]. Of note, delayed hepatotoxicity can be seen in 13% of patients following SIRT, especially in patients with a tumor burden of >50% and cirrhosis [148].

## 12. Outcomes

### 12.1. Imaging Response Criteria

The main goal of treatment is to improve the survival of patients with cancers, and, therefore, it is important to detect the change in tumor burden in patients undergoing treatment, as it has been found to be associated with increased survival. The tumor burden is evaluated with the help of standard response criteria, which require critical interrogation and cross-verification. A number of criteria have been developed to assess the treatment response in patients undergoing treatment [149,150,151]. These criteria involve one-, two-, and three-dimensional methods that can be used to measure the response after treatment [152]. Each of these methods is further subdivided into two or more categories based on the ratio of total size to the size of the enhancing component. During the Response Evaluation Criteria in Solid Tumors (RECIST) measurements, the sum of the longest one-dimensional diameter is evaluated tumor response; however, now the commonly used criteria are the modified RECIST (mRECIST) criteria, which measure the changes in tumor enhancement that can serve as a biomarker for the tumor viability [153,154]. The World Health Organization (WHO) has introduced an evaluation system for solid liver tumors in which the diameters of the tumor lesions are measured and then multiplied to evaluate the change in lesion size. In contrast to the WHO evaluation system, the European Association for the Study of the Liver (EASL) recommends measuring changes in contrast uptake of the tumor tissue to measure tumor response [150,155]. However, the criteria based on one- or two-dimensional methods are flawed due to the lack of reproducibility and inaccuracy when assessing necrotic or heterogeneous tumor lesions [156]. Due to these limitations, three-dimensional quantitative image analysis techniques have been developed, which are said to provide a reproducible assessment that is biologically accurate and provides a more clinical predictable tumor evaluation [157]. Volumetric analysis (vRECIST) includes the measurement of whole tumor volume, and therefore the change here reflects the true extent and distribution of the tumor tissue [94,158]. The quantitative EASL (qEASL) assesses changes in enhancing tumor volume and therefore goes beyond the boundaries calculated in vRECIST [94,157,159,160]. The PET Response Criteria in Solid Tumors (PERCIST) is another set of response assessment criterion developed to assess tumor response by using PET scans [161]. A liver-tissue classification using 3D multi-parameter MRI-based deep neural networks has been developed to better delineate the liver tissue for better TACE targeting has shown promising results [162]. An artificial intelligence concept using the qEASL criteria was developed to predict TACE response (overall accuracy of 78%) [163]. A study conducted by Miszczuk et al. reports that lipiodol can be a potential imaging biomarker after cTACE for tumor response. Patients with a higher overall lipiodol coverage showed higher response rates (*p* < 0.05) upon 30-day follow-up imaging studies [164,165,166,167]. Although the mainstay of tumoral response evaluation is currently the assessment of imaging response (such as with mRECIST) and chemical biomarker response (AFP), precision-medicine-based studies have recently gained momentum, thus allowing clinicians to individualize treatment plans based on genomics [168]. For example, a recent genome-based study looking at patients who underwent TACE for HCC showed that an increased PMK2 expression post-TACE was associated with attenuated survival [169].

### 12.2. Overall Survival, Progression-Free Survival, and Hepatic Progression-Free Survival

Overall survival (OS) is the most commonly used primary endpoint in oncology and clinical trials reporting results utilizing first- and second-line systemic therapies to treat advanced HCC [170]. However, other surrogate endpoints have been identified, such as objective response rate (ORR) and progression-free survival (PFS), which have helped better defined clinical outcomes and have led to accelerated regulatory approval of drugs and therapies [171,172]. OS is defined as the time after the treatment of a disease for which the patient lives. PFS is the length of time during and after the treatment of a disease when the patient is alive and the disease has not worsened [170]. In a clinical trial, measuring PFS is one way of determining how well a new treatment works and how durable its treatment effect is [170]. Likewise, hepatic progression-free survival (HPFS) is calculated from the first regional treatment until the first date of documented progression in the liver and serves as a surrogate of treatment [173].

TAE has been investigated in multiple studies, and 1-year reported overall survival of patients with nonresectable HCC ranges from 42 to 86%, whereas 2-year OS ranges from 25 to 51% [174,175,176,177,178,179]. In a meta-analysis conducted by Marelli et al., TAE was compared to TACE, and no survival benefit was seen in patients who underwent TACE compared to those who underwent TAE [130]. The use of smaller calibrated microspheres has been shown to lead to better outcomes by causing improved tumor-control rates. The use of 300–500 μm beads had an OS of 15.1 months compared to 11.1 months in patients who underwent TAE with 500–700 μm beads [180]. In terms of recurrent HCC, the use of TAE has shown to have a median OS of 46 months, with 1-, 2-, and 5-year survival rates of 86%, 74%, and 47%, respectively [181].

TACE has been shown to improve the OS in patients with unresectable HCC compared to best supportive care in patients with well-preserved liver function [178,182]. The most extensive series of TACE has been reported from Japan, which studied the outcomes of 8510 patients undergoing TACE [183]. In this series, the median OS was 34 months [183]. In the first head-to-head trial comparing TACE with DEB–TACE, no difference in median OS was found between the two groups [184]. Subsequently, multiple randomized controlled trials have been performed to compare TACE and DEB–TACE; however, no significant difference in OS has been found [184,185,186]. Studies have also reported no significant difference in OS or PFS based on the chemotherapeutic regimen used for TACE [187,188,189]. Per the TACTICS trial, sorafenib combined with TACE significantly increases the PFS as compared to TACE alone in unresectable HCC (25.2 vs. 13.5 months, *p* = 0.006) [190]. The presence of portal vein thrombosis is not a contraindication to TACE. A study evaluating the safety of TACE in patients with portal vein thrombosis showed no evidence of TACE-related hepatic infarction or acute liver failure and a 0% 30-day mortality rate [191].

In the largest retrospective series of 345 HAI procedures, patients undergoing the procedure had significantly worse outcomes if they had more advanced disease [192]. The median OS was found to be 28.6 months in another study where 253 HAI procedures were investigated [193]. However, HAI has shown to have a lower OS rates when compared to TACE [194].

Patients undergoing SIRT with unresectable HCC have a median OS of up to 20.5 months [195,196,197,198,199]. In patients with unresectable HCC without portal vein thrombosis, SIRT and TACE have been shown to have a similar median OS rate (20.5 months vs. 17.5 months, respectively) [199]. In patients with portal vein thrombosis, the use of SIRT has shown to have a median OS ranging from 5.6 to 17 months depending on the extent of portal vein involvement, functional status of the patient, and underlying liver dysfunction [195,196,198,200]. The use of SIRT compared to sorafenib in patients with advanced HCC has not been shown to have improved outcomes [201,202]. Studies reporting outcomes after SIRT segmentectomy have reported significant improvements in time-to-progression, complete response rate, local tumor control, and PFS compared to TACE [112,203,204,205]. More recently, a multiregional study comparing SIRT with TACE found no significant difference between the two in downgrading the disease prior to liver transplantation [206]. The outcomes of radiation segmentectomy have been shown to be similar to ablation and surgical resection in patients with tumors of 3 cm or smaller [203].

In a meta-analysis including 55 randomized controlled trials comparing the effectiveness of different transarterial therapies in patients with HCC, all the embolization techniques provided a significant increase in survival when compared to the best supportive care. However, there was no significant increased benefit of TACE, DEB–TACE, SIRT, or TACE combined with systemic therapy when compared to BE alone. The combination of TACE with radiotherapy or local ablation showed the best survival. SIRT was found to cause the fewest side effects, whereas patients receiving TACE combined with systemic therapy had the highest number of reported complications [207].

## 13. New Developments and Future Directions

### 13.1. Newer Drug-Eluting Beads

Drug-eluting beads (DEBs) are beads loaded with different chemotherapeutic agents. A number of different DEBs have been successfully developed and have been used in the management of hypervascular HCCs [208,209]. These beads are composed of biocompatible polymers, which can bind chemotherapeutic agents after the formation of covalent bonds [210]. As pharmacokinetics of these DEBs are better studied and further refined, the ability to deliver chemotherapeutics more efficiently and more effectively will also undoubtedly improve, potentially improving outcomes while also decreasing side effects and complications. The therapeutic potential of DEBs in precision oncology is perhaps one of the most exciting areas of current and future development in interventional oncology.

### 13.2. Radiopaque Beads

In recent years, imageable radiopaque beads have been developed, which are made radiopaque by incorporating a radio-absorber, such as iodine, zinc, tantalum, bismuth, or barium, into the beads’ manufacturing process [211,212,213,214,215]. Radiopaque beads provide real-time feedback during the embolization procedure and provide invaluable information during follow-up imaging [216,217]. A new investigational radiopaque SIRT embolic has been developed with a radiopaque bland glass microsphere which has shown strong fluoroscopic and CBCT radiopacity in preclinical testing. The use of these microspheres will allow for intra-procedural real-time confirmation of tumor targeting [218].

### 13.3. Immunoembolization

Immunoembolization is an exciting new area which involves the delivery of systemic immunotherapy agents such as granulocyte-macrophage colony-stimulating factor (GM-CSF) into the arteries supplying the HCC [219]. Immunoembolization is hypothesized to work by producing necrosis of the HCC tumor cells, thus leading to the stimulation of the antigen-presenting cells (APCs) on the tumor surface. These APCs can then increase the update of tumor antigens, which are released from the necrotic tumor cells, and lead to activation of the T cells present in the tumor microenvironment [220,221,222,223]. The development of this inflammatory response can lead to destruction of the tumor cells which did not undergo necrosis during the initial embolization. Additionally, local immune system stimulation can generate a systemic immune response against the tumor cells, which can lead to suppression of extrahepatic metastasis and prevent further tumor growth and dissemination [224,225].

### 13.4. Nanoparticles

A lot of focus in the recent years has been put into the development of nanoparticles that can be used for the diagnosis and treatment of patients with HCC [226]. The introduction of novel theranostic nanoparticles into the interventional radiology field can help diagnose, localize, and stage disease. Theranostic nanoparticles may also provide invaluable information regarding treatment response [227,228]. Such nanoparticles are designed to carry therapeutic agents to the tumor via molecular and/or external stimuli [229,230,231].

### 13.5. Combination with Systemic Therapies

The use of systemic therapies in combination with transarterial embolization techniques is an area of significant clinical and research focus [232]. Some of the many targeted systemic therapies that have been studied include, but are not limited to, MAPK Inhibitors, including sunitinib, sorafenib, imatinib, cabozantinib, lenvatinib, and selumetinib [233,234]. Others include immunotherapeutic agents such as ipilimumab, nivolumab, and pembrolizumab [235,236,237]. A majority of the studies have shown positive results, with improved outcomes and acceptable safety profiles [238].

### 13.6. Combination with Percutaneous Therapies

Combining embolization techniques with ablation for hepatocellular may enhance the therapeutic benefit of each and result in improved patient survival [239]. A majority of studies have demonstrated the safety and efficacy of a two-step or single-session transarterial and percutaneous ablation treatment for unresectable hepatic metastases, specifically for lesions >3 cm in diameter [240].

### 13.7. Pre-Operative TACE

TACE can be used as neoadjuvant therapy for the treatment of large HCCs prior to surgical resection [241]. Pre-operative TACE can also facilitate curative resection in patients with large HCCs who are otherwise not suitable candidates for liver resection [242,243]. However, the outcomes for the use of pre-operative TACE are mixed, with some studies showing improved survival outcomes and others not [244,245,246].

## 14. Conclusions

Transarterial therapies remain an important and increasingly utilized therapeutic option in the management of patients with HCC. They are unique, as they provide highly effective tumor control while preserving normal liver parenchyma and reducing toxicity. Over the last couple of decades, the evidence to support the use of transarterial therapies for managing HCC has grown to where they are now within commonly utilized treatment guidelines. A multidisciplinary approach for selecting appropriate patients and tumors is critical to optimizing clinical outcomes and technical success of the various transarterial therapies that are currently available. Advances in therapeutics, pharmacokinetics, drug delivery, and imaging will all serve to further advance transarterial therapies for HCC in the future.

## Figures and Tables

**Table 1:** Barcelona clinic liver cancer classification for the prognosis and treatment of hepatocellular carcinoma [5].

Stages	Characteristics
Very early stage (0)	Single lesion ≤2 cm Preserved liver function Performance status: 0 (fully active)
Early stage (A)	Single lesion or ≤3 nodules each ≤3 cm in size Preserved liver function Performance status: 0
Intermediate stage (B)	Multinodular Preserved liver function Performance status: 0
Advanced stage (C)	Portal invasion and/or extrahepatic spread Preserved liver function Performance status: 1 (cannot do heavy physical work) or 2 (up and about more than half the day, can look after self but cannot work)
Terminal stage (D)	Any tumor burden End-stage liver function Performance status: 3 (in bed or a chair for more than half the day and need help to look after self) or 4 (in bed or chair all the time needing complete care)

**Table 2:** Eastern Cooperative Oncology Group Performance Status Scale.

Grade	ECOG Performance Status
0	Fully active
1	Cannot do heavy physical work
2	Up and about more than half the day, can look after self but cannot work
3	In bed or a chair for more than half the day and need help to look after self
4	In bed or chair all the time needing complete care
5	Dead
6	Fully active

**Table 3:** Child–Pugh classification.

Parameter	Points Assigned		
	1	2	3
Ascites	Absent	Slight	Moderate
Serum bilirubin	<2 mg dL^−1^ (<34.2 micromol L^−1^)	2 to 3 mg dL^−1^ (34.2 to 51.3 micromol L^−1^)	>3 mg dL^−1^ (>51.3 micromol L^−1^)
Serum albumin	>3.5 mg dL^−1^ (35 g L^−1^)	2.8 to 3.5 g dL^−1^ (28 to 35 g L^−1^)	<2.8 g dL^−1^ (<28 g L^−1^)
Prothrombin time or INR	<4 or <1.7	4 to 6 or 1.7 to 2.3	>6 or >2.3
Encephalopathy	None	Grade 1 to 2	Grade 3 to 4

**Table 4:** Albumin–bilirubin (ALBI) score.

ALBI Grade	Score
Grade 1	≤2.6
Grade 2	>2.6 to 1.39
Grade 3	>1.39

**Table 5:** Platelet–ALBI (pALBI) score.

pALBI Grade	Score
Grade 1	≤−2.53
Grade 2	>−2.53 and ≤−2.09
Grade 3	>−2.09
